# On the Treatment of Vesico-Vaginal Fistula

**Published:** 1853-12

**Authors:** 


ART. VII. — *On the Treatment of Vesico-vaginal Fistula.* By J. Marion Sims, M. D., New York.

This little monograph is a concise account of Dr. S.'s method of operating for this intractable surgical lesion. It was originally published in the American Journal of the Medical Sciences, in January, 1852.

Dr. S. has, throughout the operation, some little matter of detail to describe, which he considers essential to success. The principal improvement which he suggests, and which he claims has given him a far better average of success than is usual to the operation, is in the use of the "clamp-suture." We will quote a paragraph from an article by Dr. Mussey, (Amer. Jour. Med. Sciences for Nov., 1853,) which describes the apparatus very intelligibly:

"Dr. Sims is well entitled to the thanks of the profession for having introduced what he calls the *clamp-suture*, in the treatment of vesico-vaginal fistula, consisting of two cylinders of silver, or lead, perforated at several points for the passage of pieces of small silver wire, which are to supply the place of thread, and which are to be prevented from slipping by perforated shot being carried down upon them, pressed against the cylinders, and kept in place by being firmly pinched with pliers. Dr. S. makes his cylinders one line in diameter, and his wires of the size of a horse hair." S. B. H.

